# A Bibliometric Analysis of the Role and Research Trending of Bronchoalveolar Lavage in the Diagnosis and Treatment of Ventilator-Associated Pneumonia

**DOI:** 10.7759/cureus.62583

**Published:** 2024-06-18

**Authors:** Shujuan Wei, Changhao Cheng, Xiaofeng Zhong

**Affiliations:** 1 Intensive Care Unit, Wuhan Pulmonary Hospital, Wuhan, CHN; 2 Tuberculosis Ward, Wuhan Pulmonary Hospital, Wuhan, CHN

**Keywords:** research dynamics, citespace, bibliometric analysis, bronchoalveolar lavage, ventilator-associated pneumonia

## Abstract

Ventilator-associated pneumonia (VAP) is one of the most common complications in intensive care units (ICUs) and negatively affects patient outcomes. Despite its widespread use as a diagnostic and therapeutic measure, the application and effectiveness of bronchoalveolar lavage (BAL) in the management of VAP require further exploration. This study aimed to evaluate the research dynamics, major trends, and scientific networks of BAL in the diagnosis and treatment of VAP using bibliometric analysis. Literature from the Web of Science database on BAL for the diagnosis and treatment of VAP from 1990 to 2024 was screened and analyzed. Keyword co-occurrence, trend analysis, and citation burst analyses were conducted using CiteSpace to identify research hotspots, core authors, institutions, and countries, as well as the evolution of research domains. The bibliometric analysis included 968 publications. Trend analysis indicated growing interest in BAL techniques, particularly in the categories of RESPIRATORY SYSTEM (burst score: 27.82) and MEDICINE, RESEARCH, and EXPERIMENTAL (burst score: 7.41). The co-citation analysis highlighted influential authors in the field, such as Torres (burst score: 9.35), Croce (burst score: 5.86), and Meduri (burst score: 5.71). Keyword analysis results revealed core clusters in the treatment of VAP with BAL, including “nonbronchoscopic lavage” (silhouette value: 0.703), “ICU-acquired infection” (silhouette value: 0.7), and “ventilator-associated tracheobronchitis” (silhouette value: 0.637). Additionally, geographic analysis showed that North America and Europe dominated the research in this field. Recently, research trends regarding protected specimen brushes and quantitative culture techniques have emerged. This study found broad applications of BAL in VAP management, especially in improving diagnostic accuracy and treatment outcomes. Optimized strategies such as improvement of lavage techniques and multidisciplinary collaboration may emerge as potential research hotspots in the future.

## Introduction and background

Ventilator-associated pneumonia (VAP) refers to pneumonia that occurs after 48 hours of mechanical ventilation and is one of the most common nosocomial infections in intensive care units (ICUs) [1,2]. According to reports from the United States Centers for Disease Control and Prevention (CDC), the incidence of VAP in ventilated patients ranges from 9% to 27%, significantly increasing the hospital stay duration, healthcare costs, and mortality rates [3]. Consequently, the prevention and treatment of VAP have become significant research topics in healthcare [4].

Bronchoalveolar lavage (BAL), a technique employed to diagnose and treat respiratory diseases, has garnered widespread attention in recent years for the diagnosis and treatment of VAP [5]. BAL involves the instillation of a small amount of saline into the alveoli, followed by the retrieval of lavage fluid for analysis to obtain deep lung samples [6]. By examining the cellular components, microbes, and other biomarkers within the lavage fluid, BAL aids in the diagnosis of VAP and provides guidance for selecting appropriate antibiotic therapy [6,7]. Although BAL has shown potential for the management of VAP, its clinical significance, optimal implementation methods, and safety remain debated in research topics.

With the rapid development of medical research, clinical studies have often struggled to answer complex medical questions. This necessitates the use of bibliometric analysis to evaluate numerous research areas comprehensively and identify research trends, knowledge structures, and future directions [8]. Bibliometrics, which applies mathematical and statistical methods to analyze scientific literature, allows for the quick identification of research hotspots and development trends within a field by analyzing publication trends, citations, regional contributions, institutional involvement, and author collaboration networks of research literature on specific topics [9]. The application of bibliometric analysis tools, such as CiteSpace, has made comprehensive analyses of scientific literature more efficient and in-depth [10]. These tools can process large-scale literature data and visually present complex scientific networks, including research hotspots, interdisciplinary intersections, core authors, and collaborations, providing researchers with profound analysis results [11].

In this study, using bibliometric methods and CiteSpace analysis software, we explored the research dynamics and trends in the field of BAL application in VAP management, offering evidence and ideas for the prevention and treatment of VAP.

## Review

Methods

Data Sources and Search Strategy

The primary data source for this study was the Web of Science (https://www.webofscience.com/wos/). The literature was searched using the following keywords and combinations: “ventilator-associated pneumonia,” “VAP,” “Bronchoalveolar Lavage,” “diagnosis,” “treatment,” and “bibliometric analysis.” The search was conducted from January 1, 1990, to March 1, 2024, and was limited to English-language publications.

Inclusion and Exclusion Criteria

The inclusion criteria were original research, review articles, and case reports related to the application of BAL for the diagnosis and treatment of VAP, which provided sufficient data for bibliometric analysis. Conference abstracts, commentaries, opinion articles, letters to the editor, and studies that were published repeatedly or had incomplete data were excluded.

Bibliometric Statistical Methods

Descriptive statistical analysis was used for quantitative data, including frequencies, percentages, means, and standard deviations, to describe the basic characteristics and outcomes of the studies. CiteSpace software was used for bibliometric analysis [10,11]. CiteSpace is information visualization software that integrates information visualization techniques, bibliometric methods, and data mining algorithms capable of detecting research hotspots and trends in scientific literature [10,11]. The “TimeSlicing” was set from January 1, 1990, to March 1, 2024, with a time slice set to one year. Nodes were designated as keywords, authors, institutions, and objects for visualization. This setup facilitated the creation of keyword co-occurrence maps, keyword clustering analyses, author collaboration networks, and research hotspots. The color legend was provided for the exact year-color correspondence. The solid nodes represent topics, with the innermost circles indicating older research or other topics and the outermost circles indicating more recent research or other topics. The color gradient, ranging from purple (1991) to red (2024), represents the year of the topics. The color of the edges and lines represents the year of publication, ranging from 1991 (purple) to 2024 (red). For cluster analysis, different color represents a different cluster. The thresholds and parameters were adjusted based on the actual volume of data and analysis objectives to ensure the results’ accuracy and interpretability. A burst refers to the frequency surge of a particular type of event, such as a research topic. 

The analysis steps included a co-citation analysis to identify key publications and benchmark articles within the research domain and a keyword co-occurrence analysis to discover research hotspots and thematic trends in the field. Author collaboration network analysis was used to identify the main research groups and core authors within each domain. A bibliographic coupling network analysis was used to evaluate the interconnectedness between different studies and the knowledge structure within the field. Temporal view analysis was used to track the evolution of research themes and hotspots over time. Cluster analysis aims to reveal sub-domains and the internal structure of research themes by analyzing clusters of literature.

Results

Literature Search and Screening Results

In this study, 1219 articles or reviews were identified through literature retrieval. The initial screening excluded 65 duplicates. Based on the inclusion and exclusion criteria, 186 articles that were neither original research nor review articles were excluded, resulting in 968 articles included in the bibliometric analysis.

Publication Trend

**Figure 1:**
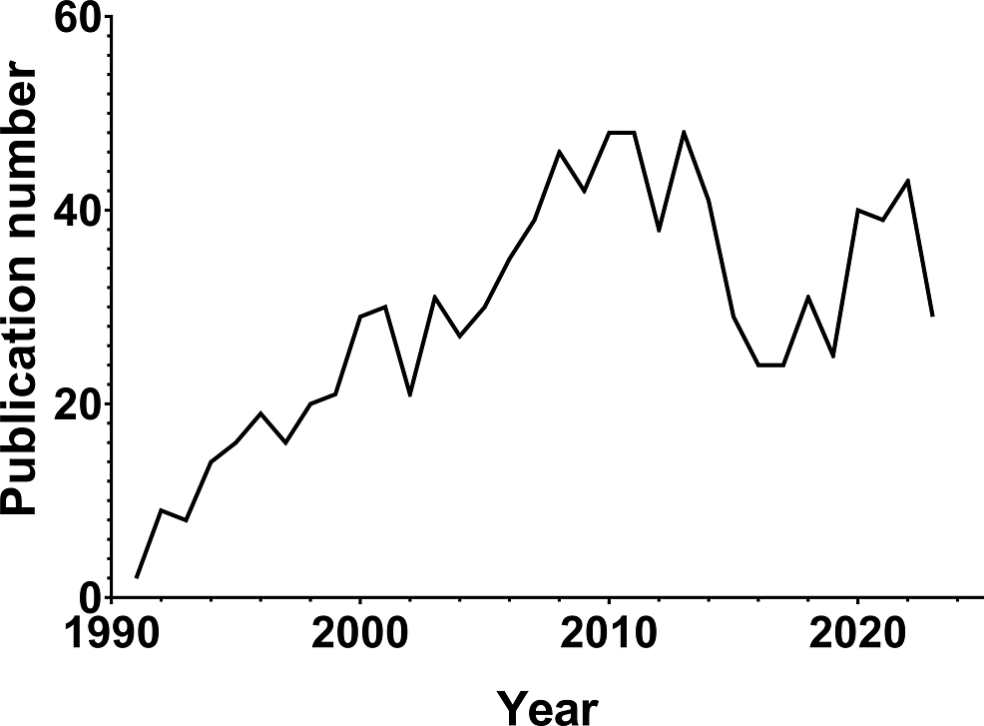
The trend chart of the number of publications by years.

The distribution of publication years highlights the interest of researchers and the evolutionary process of employing BAL for the diagnosis and treatment of VAP. Since the introduction of the BAL technique in 1990, research has been conducted worldwide. Among relevant research articles, 968 were identified. Publication volume showed an ascending trend prior to 2010, peaked in 2010, and then gradually declined (Figure 1).

Cooperation Network Analysis

**Figure 2:**
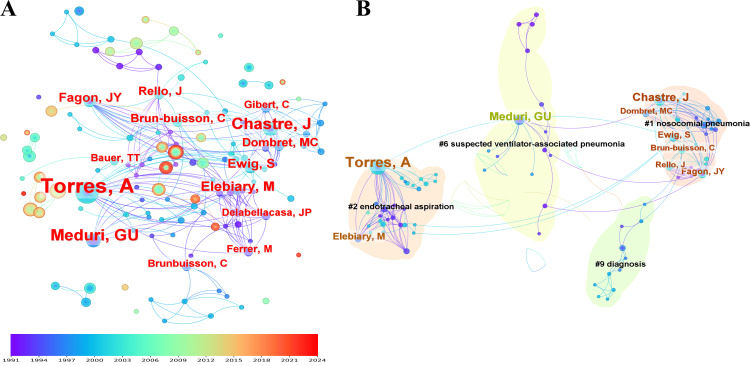
The bibliometric analysis of authors in the field of bronchoalveolar lavage in the diagnosis and treatment of ventilator-associated pneumonia. A, Author details; B, Major cluster by author. The solid nodes represent topics, with the innermost circles indicating older ones and the outermost circles indicating more recent ones. The color gradient, ranging from purple (1991) to red (2024), represents the publication year of the research topics.

Author: As illustrated in the graph, the cooperative network among authors features nodes that represent individual authors with larger font sizes, indicating higher publication volumes. The connections between nodes signify collaborative relationships among the authors (Figure 2). The top-ranked authors were Torres et al. [12], with a citation count of 24, followed by Goldberg et al. [13] (citation count: 17), Fabian [14] (citation count: 15), Luyt [15] (citation count: 13), and Swanson et al. [16] (citation count: 12) (Figure 2A). The network comprises four distinct clusters. Cluster #1, which focused on nosocomial pneumonia, included 29 members and had a silhouette value of 0.989, indicating a highly cohesive cluster. The second-largest cluster, #2, also comprised 29 members but with a silhouette value of 0.971 and was centered on VAP. The third largest cluster, with 15 members, had a silhouette value of 0.964 and was labeled suspected VAP. The fourth largest, comprising 11 members with a silhouette value of 0.954, focused on diagnosis (Figure 2B).

In the analysis of burstiness, Torres [12] was identified as the leading author in the 1990s, exhibiting a burstiness measure of 9.35. with Goldberg [13] exhibiting a measure of 5.86, and Meduri [17] with 5.71. Fabian [16] and Papazian [18] rounded out the top five with burstiness measures of 5.00 and 4.63, respectively (Table 1).

**Table 1:** Top 10 authors with the strongest citation bursts.

Authors	Emergence year	Strength	Begin year	End year
Meduri GU	1992	5.71	1992	1998
Chastre J	1992	4	1992	2003
Torres A	1993	9.35	1993	2003
Elebiary M	1993	3.99	1993	1997
Croce MA	1995	3.82	2003	2006
Goldberg	2008	5.86	2008	2015
Fabian, Timothy C	2008	5	2008	2015
Swanson, Joseph M	2008	3.89	2008	2018
Papazian, Laurent	2009	4.63	2009	2012
Magnotti, Louis J	2009	4.52	2009	2018

**Figure 3:**
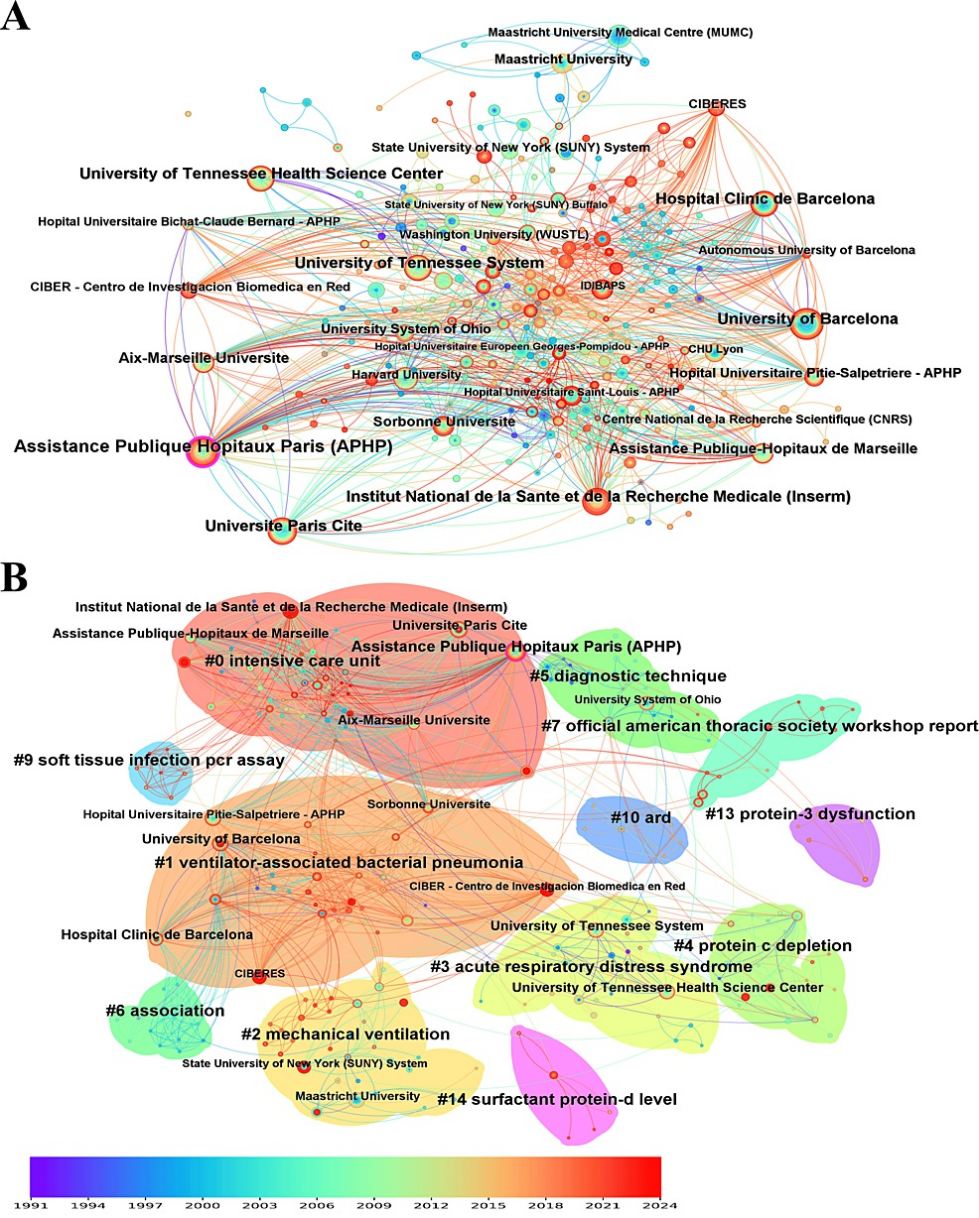
The bibliometric analysis of institutions in the field of bronchoalveolar lavage in the diagnosis and treatment of ventilator-associated pneumonia. A, institution co-operation network; B, institution co-operation cluster. The solid nodes represent topics, with the innermost circles indicating older ones and the outermost circles indicating more recent ones. The color gradient, ranging from purple (1991) to red (2024), represents the publication year of the topics.

Institution: In an institutional cooperation network, each node represents an institution, with the lines between nodes indicating collaborative ties. The size of the central node correlated with the volume of publications from that institution (Figure 3). The top five institutions ranked by citation counts were the Assistance Publique Hopitaux Paris (APHP), with 77 citations. This was followed by the University of Barcelona, with 48 citations. The Hospital Clinic de Barcelona ranked third with 45 citations. The Institut National de la Sante et de la Recherche Medicale (Inserm) and Universite Paris Cite were tied for fourth, each with 44 citations (Figure 3A). The top five primary clusters spanning the 1990s to 2024 were identified as shown in Figure 3B.

In the burst analysis, the University of Barcelona emerged as the top institution with a burst score of 8.51. The Hospital Clinic de Barcelona followed with 7.15. The Institut National de la Sante et de la Recherche Medicale (Inserm) ranked third, scoring 5.12. The Maastricht University Medical Centre (MUMC) was fourth with a score of 4.82, and the University of California System ranked fifth with 4.61 (Table 2).

**Table 2:** Top 15 institutions with the strongest citation bursts.

Institutions	Emergence year	Strength	Begin year	End year
Hopital Universitaire Bichat-Claude Bernard - APHP	1992	3.59	1992	2004
University of Barcelona	1993	8.51	1993	2003
Hospital Clinic de Barcelona	1993	7.15	1993	2003
Maastricht University Medical Centre (MUMC)	1995	4.82	1995	2001
Hopital Universitaire Europeen Georges-Pompidou - APHP	1992	3.89	2002	2007
Universite Paris Cite	1992	3.73	2002	2004
State University of New York (SUNY) Buffalo	2002	4.23	2004	2011
State University of New York (SUNY) System	1995	4.47	2007	2011
University of California System	2008	4.61	2008	2011
University of California San Francisco	2008	4.36	2008	2011
Centre National de la Recherche Scientifique (CNRS)	2003	4.34	2009	2016
Institut National de la Sante et de la Recherche Medicale (Inserm)	1995	5.12	2012	2022
CIBERES	2008	4.57	2014	2024
CIBER - Centro de Investigacion Biomedica en Red	2008	4.57	2014	2024
Catholic University of the Sacred Heart	2013	3.79	2016	2021

The top five primary clusters spanning the 1990s to 2024 were identified (Figure 3B). The largest cluster (#0) included 63 members and had a silhouette value of 0.927, focusing on ICUs. The second largest cluster (#1) comprised 44 members with a silhouette value of 0.911, concentrating on ventilator-associated bacterial pneumonia. The third largest cluster (#2) had 32 members and a silhouette value of 0.907 and was dedicated to mechanical ventilation. The fourth largest cluster (#3) included 27 members with a silhouette value of 0.915 and focused on acute respiratory distress syndrome. The fifth-largest cluster (#4) contained 24 members and had a silhouette value of 0.873, centered on protein C depletion.

Analysis of the institutional cooperation network via CiteSpace revealed that the Assistance Publique Hopitaux Paris, Institut National de la Sante et de la Recherche Medicale, Universite Paris Cite, and other institutions played central roles in VAP research, demonstrating a network of collaboration between scientific institutions. These institutions span multiple countries, including France, Spain, and the United States, highlighting the significance of international cooperation (Figure 3).

**Figure 4:**
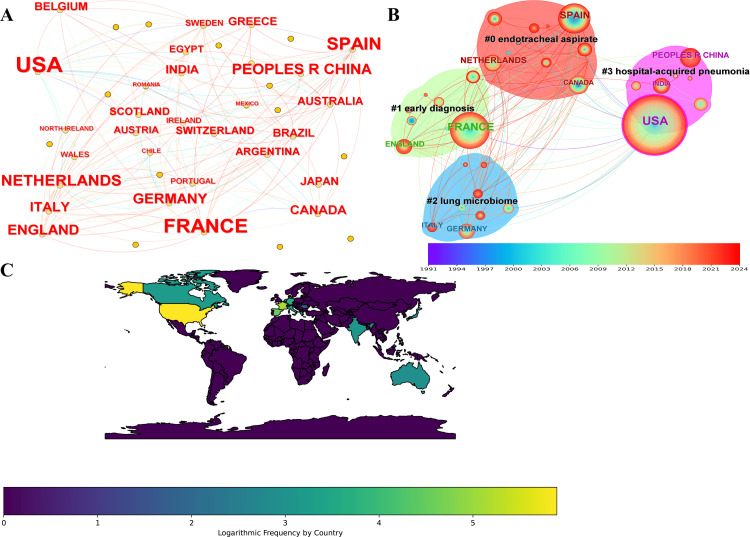
The bibliometric analysis of country in the field of bronchoalveolar lavage in the diagnosis and treatment of ventilator-associated pneumonia. A, country co-operation network; B, country co-operation cluster; C, distribution of publication citations in world map model. The color gradient, ranging from purple (1991) to red (2024), represents the publication year of the topics.

Country: The cooperation network among the countries is depicted in Figure 4, where the font size represents the volume of publications. The literature originated from 55 countries, with the United States (362 articles, 31.8% of the total), France (171 articles, 15.0%), and Spain (92 articles, 8.0%) being the top three contributors. In recent years, 46 publications indexed by the Web of Science originated in China-the country’s involvement in VAP-related research related to VAP was on the rise (Figure 4A). This trend was also evident in the burst analysis, in which China had a burst value of 11.81, followed by Spain at 11.69, Germany at 4.49, France at 4.16, and Turkey at 3.65. The network analysis identified five major clusters. The largest cluster (#0) comprised 10 members with a silhouette value of 0.646, focusing on the endotracheal aspirate. The second largest cluster (#1), with eight members and a silhouette value of 0.434, pertained to early diagnosis. The third largest cluster (#2) also had eight members but with a silhouette value of 0.625 and was dedicated to the lung microbiome. The fourth largest cluster (#3), comprising seven members, had a silhouette value of 0.541 and focused on hospital-acquired pneumonia. The fifth largest cluster (#4), with three members, had a high silhouette value of 0.937, centering on colonization infection (Figure 4B). In the centrality analysis (Figure 4C), the United States ranked first with a centrality of 0.18, followed by France and Spain at 0.10, Brazil at 0.10, and England at 0.09.

Analysis of the international cooperation network revealed that research related to VAP was primarily concentrated in North America, Europe, and some Asian countries, particularly in the United States, Spain, Germany, and China. These countries have published a significant volume of literature on VAP and have exhibited a trend toward transnational collaboration.

Co-occurrence Network Analysis

**Figure 5:**
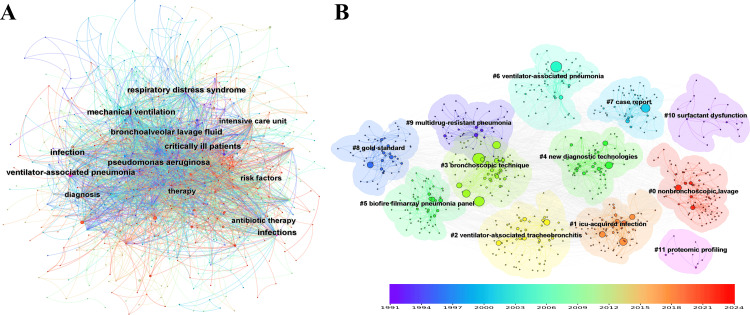
The bibliometric analysis of keywords in the field of bronchoalveolar lavage in the diagnosis and treatment of ventilator-associated pneumonia. A, keywords distribution network; B, keywords cluster. The color gradient, ranging from purple (1991) to red (2024), represents the publication year of the topics.

Keywords: CiteSpace analysis of keyword co-occurrence generated a network of 673 nodes and 3335 links, resulting in a network density of 0.0147. Each node represents a keyword, and the links indicate co-occurrence relationships, with larger font sizes denoting a higher frequency of occurrence. As shown in Figure 5A, the keywords with the highest citation count were “bronchoalveolar lavage,” with 438 citations. The second highest was “ventilator associated pneumonia,” with 380 citations, followed by “nosocomial pneumonia” with 305 citations. The fourth was “ventilator-associated pneumonia,” with 248 citations, and the fifth was “diagnosis,” with 187 citations. The network comprised 12 clusters (Figure 5B).

The largest cluster (#0) had 80 members and a silhouette value of 0.703, labeled as “nonbronchoscopic lavage.” The most cited members in this cluster were “mortality,” “respiratory distress syndrome,” and “acute lung injury.” The second largest cluster (#1) had 76 members and a silhouette value of 0.7, labeled as “ICU-acquired infection.” The most cited members in this cluster were “intensive care unit,” “risk factors,” and “bronchoalveolar lavage fluid.” The third largest cluster (#2) had 72 members and a silhouette value of 0.637, labeled as “ventilator-associated tracheobronchitis.” The most cited members in this cluster were “impact,” “antibiotic therapy,” and “outcome.” The fourth largest cluster (#3) had 71 members and a silhouette value of 0.819, labeled as “bronchoscopic technique.” The most cited members in this cluster were “bronchoalveolar lavage,” “nosocomial pneumonia,” and “diagnosis.” The fifth largest cluster (#4) had 68 members and a silhouette value of 0.774 and was labeled “new diagnostic technologies.” The most cited members in this cluster were “mechanical ventilation,” “infection,” and “accuracy.” The results of the keyword co-occurrence network indicated that high-frequency co-occurrence of keywords such as “VAP,” “bronchoalveolar lavage,” “diagnosis,” and “treatment” revealed research hotspots and trends.

The top-ranked keyword by bursts was “protected specimen brush” in cluster #3, with bursts of 33.55. The second was “quantitative culture techniques” in cluster #3, with bursts of 16.35. The third was “bacterial pneumonia” in cluster #3, with bursts of 15.97. The fourth was “pulmonary infections” in cluster #3, with bursts of 11.80. The fifth was “intubated patients” in cluster #3, with bursts of 11.73. The sixth was “continuous mechanical ventilation” in cluster #3, with bursts of 11.20. The seventh was “nosocomial bacterial pneumonia” in cluster #3, with bursts of 10.61. The eighth was “telescoping plugged catheter” in cluster #3, with bursts of 9.76. The ninth was “quantitative cultures” in cluster #3, with bursts of 9.71. The tenth was “infectious diseases society” in cluster #9, with bursts of 8.83 (Table 3).

**Table 3:** Top 25 keywords with the strongest citation bursts.

Keywords	Emergence year	Strength	Begin year	End year
protected specimen brush	1991	33.55	1991	2002
quantitative culture techniques	1991	16.35	1991	1999
bacterial pneumonia	1991	15.97	1991	2002
pulmonary infections	1991	11.8	1991	2002
intubated patients	1991	11.73	1991	2003
continuous mechanical ventilation	1991	11.2	1991	2000
fiberoptic bronchoscopy	1991	5.83	1991	1999
nosocomial bacterial pneumonia	1992	10.61	1992	2002
telescoping plugged catheters	1992	9.76	1992	2002
bacteriologic diagnosis	1992	6.36	1992	1999
catheter	1992	5.13	1992	2001
suspected pneumonia	1996	5.89	1996	2002
bronchoscopic techniques	1996	5.57	1996	2002
quantitative cultures	1998	9.71	1998	2006
pulmonary infection score	2006	7.94	2006	2016
outcm	2006	7.89	2008	2018
c reactive protein	2006	5.19	2008	2019
resistance	2009	5	2009	2020
procalcitonin	2010	5.72	2010	2020
pneumonia	2005	6.2	2011	2024
infections	1992	4.8	2012	2015
identification	2008	4.78	2014	2022
epithelial lining fluid	2007	5.14	2017	2024
guidelines	1994	4.94	2018	2024
infectious diseases society	2019	8.83	2019	2024

This section also calculates the centrality and sequence of occurrences of the paper keywords. Keywords with high centrality ( > 0.1) were considered significant within the network. The top-ranked item by centrality was “respiratory distress syndrome” in cluster #0, with a centrality of 0.17. The second was “critically ill patients” in cluster #1, with a centrality of 0.13. The third was “infection” in cluster #4, with a centrality of 0.13. The fourth was “mechanical ventilation” in cluster #4, with a centrality of 0.11. The fifth was “Pseudomonas aeruginosa” in cluster #6, with a centrality of 0.11. The sixth was “bronchoalveolar lavage fluid” in cluster #1, with a centrality of 0.10. The seventh was “infections” in cluster #3, with a centrality of 0.10. The eighth was “ventilator-associated pneumonia” in cluster #7, with a centrality of 0.10. The ninth was “risk factors” in cluster #1, with a centrality of 0.09. The tenth was “antibiotic therapy” in cluster #2, with a centrality of 0.08.

**Figure 6:**
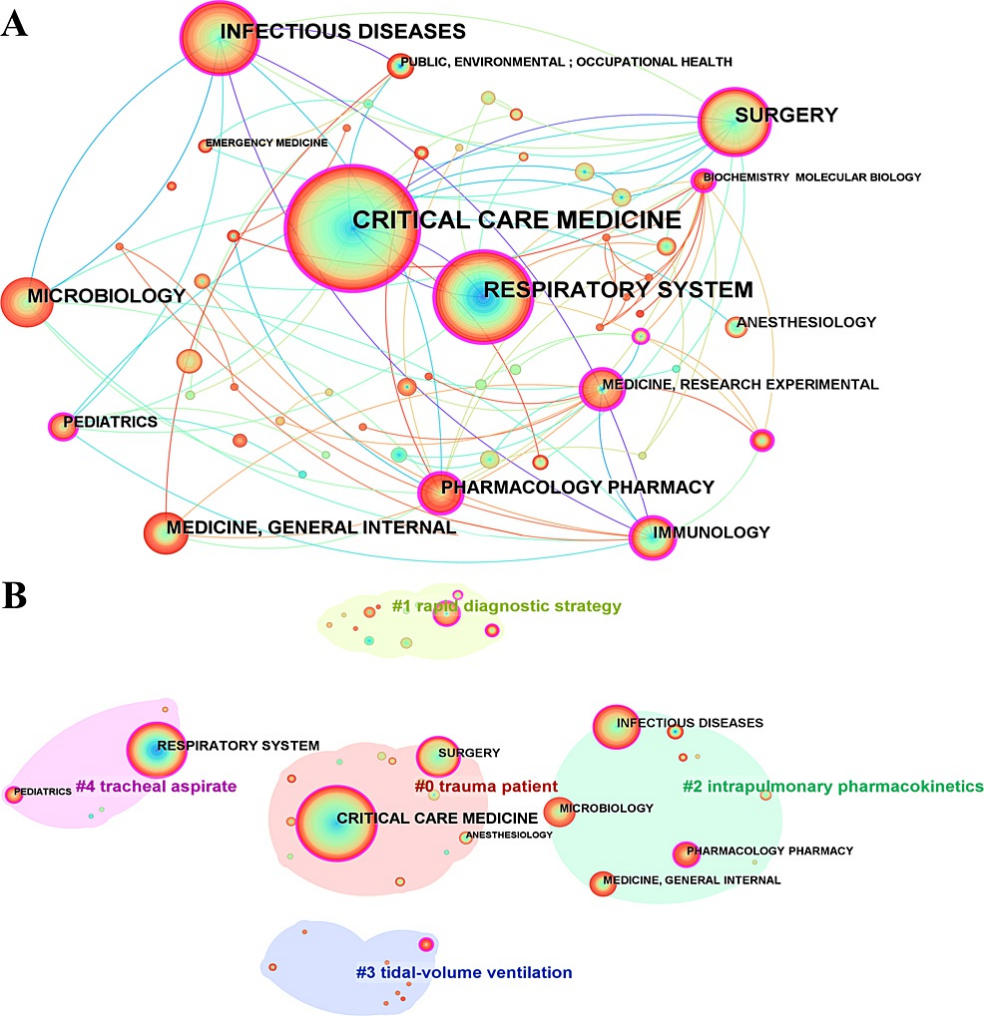
The bibliometric analysis of category in the field of bronchoalveolar lavage in the diagnosis and treatment of ventilator-associated pneumonia. A, category distribution network; B, category cluster. The color gradient, ranging from purple (1991) to red (2024), represents the publication year of the topics.

Category: An analysis of the disciplinary categorization of the literature included in the study revealed the following ranking based on citation counts: CRITICAL CARE MEDICINE emerged as the top category, with 392 citations. The following were RESPIRATORY SYSTEM with 220 citations, INFECTIOUS DISEASES with 152 citations, SURGERY with 129 citations, and MICROBIOLOGY with 109 citations (Figure 6A). The network comprises six major clusters (Figure 6B).

The largest cluster (#0) included 13 members and had a silhouette value of 0.672, labeled trauma patient care. The second-largest cluster (#1) comprised 12 members with a silhouette value of 0.861 and was labeled as a rapid diagnostic strategy. The third largest cluster (#2) featured nine members with a silhouette value of 0.848 and focused on intrapulmonary pharmacokinetics. The fourth largest cluster (#3) had eight members with a silhouette value of 0.925 and was labeled tidal-volume ventilation. The fifth-largest cluster (#4) comprised five members and had a silhouette value of 0.937 based on tracheal aspirate analysis. The sixth largest cluster (#5) included three members with a silhouette value of 0.928, labeled post-severe traumatic brain injury care.

In terms of burst analysis, the RESPIRATORY SYSTEM category in cluster #4 led to 27.82. This was followed by MEDICINE, RESEARCH, and EXPERIMENTAL in cluster #1 with bursts of 7.41, PHARMACOLOGY and PHARMACY in cluster #2 with bursts of 6.82, SURGERY in cluster #0 with bursts of 4.20, and MEDICINE, GENERAL, and INTERNAL in cluster #2 with bursts of 4.19.

The centrality rankings were led by MEDICINE, RESEARCH, and EXPERIMENTAL in cluster #1, with a centrality of 0.39. BIOCHEMISTRY & MOLECULAR BIOLOGY in cluster 3 had a centrality of 0.37, followed by RESPIRATORY SYSTEM in cluster 4 with a centrality of 0.28, CRITICAL CARE MEDICINE in cluster 0 with a centrality of 0.24, and PHARMACOLOGY & PHARMACY in cluster 2 with a centrality of 0.20.

This analysis indicates that in terms of technological innovation and application, the study of genomics, proteomics, and biomarkers is becoming a new hotspot in VAP research. Regarding the development of prevention strategies, as an understanding of the pathogenesis of VAP deepens, research into prevention strategies becomes a new focus. Effective prevention measures identified through keyword co-occurrence analysis included hygiene management, early diagnosis, and customized treatment plans.

Co-citation Analysis

Reference: The co-citation network of references generated 1317 nodes and 4160 links, with a network density of 0.0048. Co-citation network analysis revealed frequent citations of works by Chastre [19], indicating these studies as seminal and foundational in the research on VAP and bronchoalveolar lavage.

**Figure 7:**
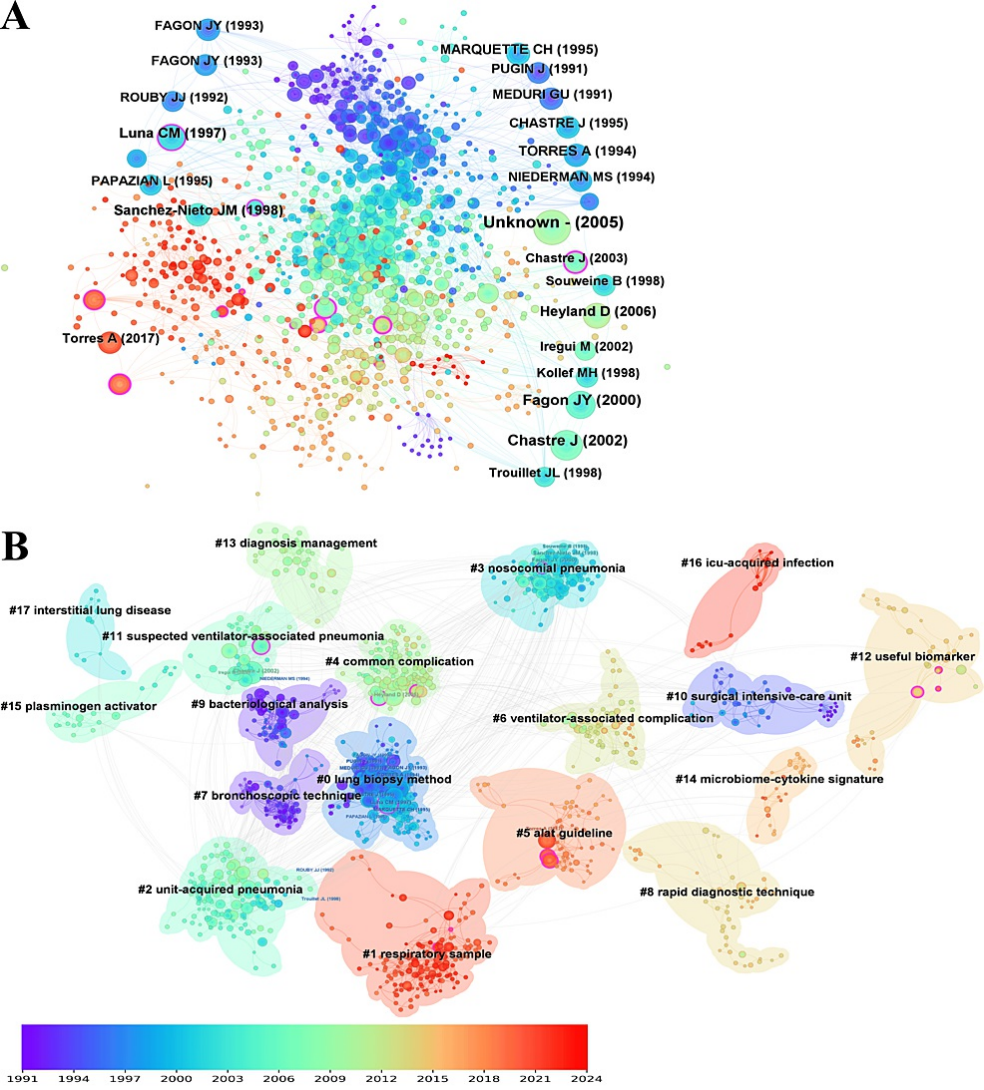
The bibliometric analysis of references in the field of bronchoalveolar lavage in the diagnosis and treatment of ventilator-associated pneumonia. A, cited reference network; B, cited reference cluster. The color gradient, ranging from purple (1991) to red (2024), represents the publication year of the topics.

The most cited reference was from the American Journal of Respiratory and Critical Care Medicine, 2005, Volume 171, Page 388 [20], with 93 citations. This was followed by Chastre 2002, in the same journal, Volume 165, Page 867 [21], with 65 citations. The third most cited was Fagon 2000, in the Annals of Internal Medicine, Volume 132, Page 621 [22], with 58 citations. Luna, 1997, in Chest, Volume 111, Page 676 [23], received 46 citations, tied with Heyland, 2006, in the New England Journal of Medicine, Volume 355, Page 2619 [24], also with 46 citations (Figure 7A). The network comprised 20 clusters (Figure 7B).

The largest cluster (#0), with 166 members and a silhouette value of 0.834, was labeled the lung biopsy method. The second-largest cluster (#1), with 146 members and a silhouette value of 0.979, focused on respiratory samples. The third largest cluster (#2), with 130 members and a silhouette value of 0.787, was centered on unit-acquired pneumonia. The fourth-largest cluster (#3), with 111 members and a silhouette value of 0.921, focused on nosocomial pneumonia. The fifth-largest cluster (#4), with 108 members and a silhouette value of 0.889, was identified as a common complication, specifically ventilator-associated pneumonia.

The top-ranked reference by burst was an article in 2005 from the American Journal of Respiratory and Critical Care Medicine, Volume 171, page 388 [20], in Cluster #11, with bursts of 45.91. The second was by Chastre (2002), in the same journal, Volume 165, Page 867 [21] in Cluster #11, with bursts of 33.61. The third was by Fagon (2000) in the Annals of Internal Medicine, Volume 132, Page 621 [22] in Cluster #3, with bursts of 29.20. The fourth was by Heyland (2006) in the New England Journal of Medicine, Volume 355, page 2619 [24], in Cluster #4, with bursts of 23.01. The fifth was by Torres (2017) in the European Respiratory Journal, Volume 50, Page 0 [25] in Cluster #5, with bursts of 21.98.

The top reference by centrality was Anand, 2009, in Chest, Volume 135, Page 641 [26], with a centrality of 0.57. The second was by Gibot, 2004, in New England Journal of Medicine, Volume 350, page 451 [27], with a centrality of 0.43. The third was by Melsen (2013) in The Lancet Infectious Diseases, Volume 13, Page 665 [28], with a centrality of 0.41. The fourth was by Chastre, 2003, in JAMA, Volume 290, Page 2588 [29], with a centrality of 0.36. The fifth was by Kalil (2016) in Clinical Infectious Diseases, Volume 63, Page E61 [30], with a centrality of 0.28.

Cited Journal

**Figure 8:**
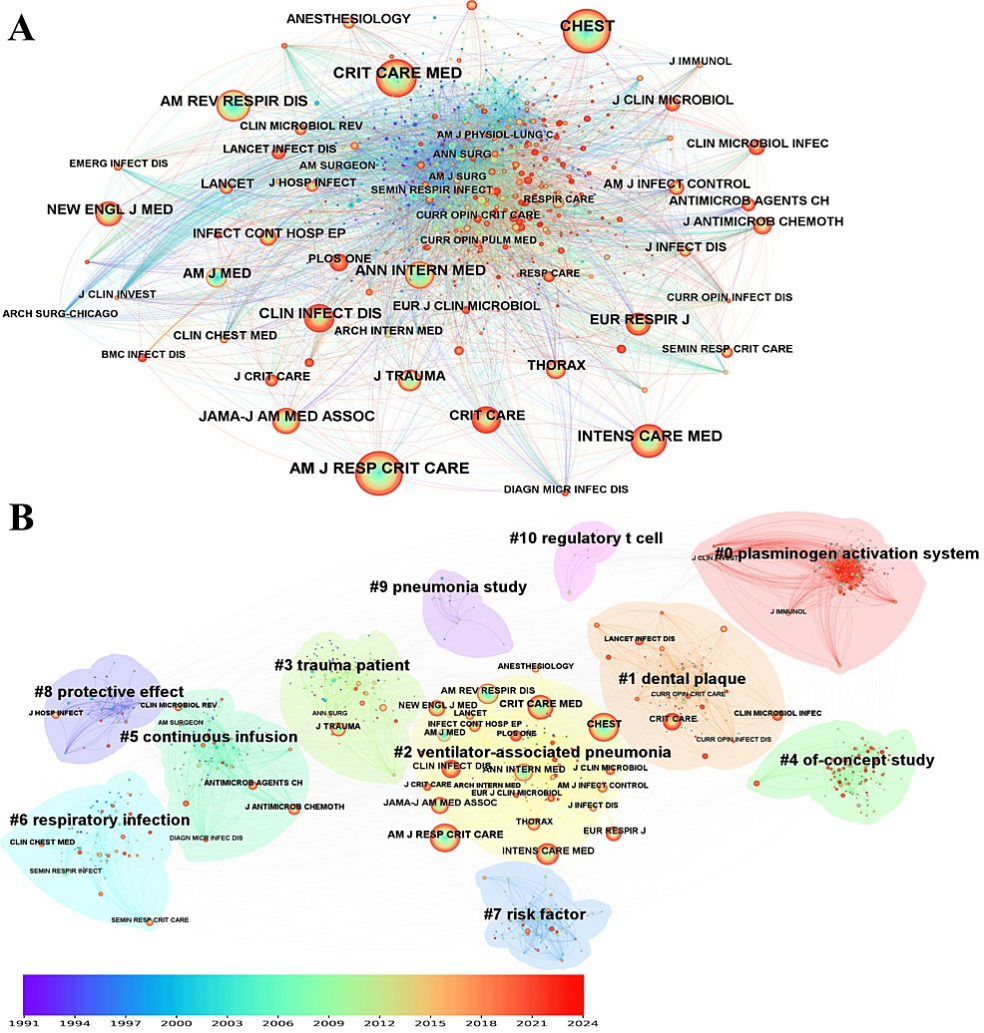
The bibliometric analysis of cited journal in the field of bronchoalveolar lavage in the diagnosis and treatment of ventilator-associated pneumonia. A, cited journal distribution network; B, cited journal cluster. The color gradient, ranging from purple (1991) to red (2024), represents the publication year of the topics.

The analysis revealed that the American Journal of Respiratory and Critical Care Medicine (AM J RESP CRIT CARE) topped the list of journals by citation counts with 756 citations. Following closely were CHEST with 753 citations, Critical Care Medicine (CRIT CARE MED) with 699 citations, Intensive Care Medicine (INTENS CARE MED) with 630 citations, and the American Review of Respiratory Disease (AM REV RESPIR DIS) with 512 citations (Figure 8A). The high citation frequencies of articles published in the American Journal of Respiratory and Critical Care Medicine, CHEST, and Critical Care Medicine (756, 753, and 699 citations, respectively) underscore their prominence as leading journals in the field of ventilator-associated pneumonia research, reflecting the high quality of their publications. The network comprised 11 clusters (Figure 8B).

The largest cluster (#0), with 137 members and a silhouette value of 0.768, was focused on the plasminogen activation system. The second largest cluster (#1), with 92 members and a silhouette value of 0.735, dealt with dental plaques. The third-largest cluster (#2), with 91 members and a silhouette value of 0.769, was dedicated to ventilator-associated pneumonia. The fourth largest cluster (#3), with 89 members and a silhouette value of 0.697, was associated with trauma patients. The fifth largest cluster (#4), with 88 members and a silhouette value of 0.636, was described as a proof-of-concept study.

Regarding bursts of citations, the American Journal of Medicine (AM J MED) led with a burst of 50.69, followed by PLOS ONE, the American Review of Respiratory Disease (AM REV RESPIR DIS), the Journal of Infectious Diseases (J INFECT DIS), and BMC Infectious Diseases (BMC INFECT DIS) with bursts of 36.27, 35.31, 21.85, and 17.31, respectively (Table 4).

**Table 4:** Top 25 cited journals with the strongest citation bursts.

Cited Journals	Emergence year	Strength	Begin year	End year
AM J MED	1991	50.69	1991	2005
AM REV RESPIR DIS	1991	35.31	1991	2004
J INFECT DIS	1991	21.85	1991	1999
ARCH INTERN MED	1991	14.07	1991	2004
AM J MED SCI	1992	10.04	1992	2003
AMERICAN REVIEW OF RESPIRATORY DISEASE	1992	9.56	1992	1997
ARCH SURG-CHICAGO	1992	9.53	1992	2006
SEMIN RESPIR INFECT	1991	16.09	1994	2007
ANN INTERN MED	1991	13.81	1994	2005
RADIOLOGY	1994	10.51	1994	2004
REV INFECT DIS	1992	9.41	1995	2006
J TRAUMA ACUTE CARE	2013	11.62	2013	2018
LANCET INFECT DIS	2006	16.67	2014	2024
ANN INTENSIVE CARE	2014	10.82	2014	2024
PLOS ONE	2010	36.27	2015	2024
BMC INFECT DIS	2011	17.31	2016	2024
CRIT CARE	1998	13.01	2016	2024
LANCET RESP MED	2017	15.76	2017	2024
CLIN MICROBIOL INFEC	1998	14.81	2017	2024
INT J INFECT DIS	2017	13.8	2017	2024
SCI REP-UK	2017	13.19	2017	2024
J INFECTION	2003	10.19	2017	2024
FRONT MICROBIOL	2018	9.65	2018	2024
OPEN FORUM INFECT DI	2019	9.39	2019	2024
BMC PULM MED	2005	10.3	2020	2024

In terms of centrality, the American Journal of Epidemiology (AM J EPIDEMIOL) in cluster #3 led with a centrality of 0.10. The Journal of Clinical Investigation (J CLIN INVEST) in cluster 0 had a centrality of 0.09, followed by the American Journal of Pathology (AM J PATHOL) in cluster #0 with a centrality of 0.08. The American Journal of Surgery (AM J SURG) in cluster #3 had a centrality of 0.06, and the British Medical Journal (BMJ) in cluster #1 had a centrality of 0.06.

Highly cited journals such as the “American Journal of Respiratory and Critical Care Medicine” and “CHEST” signify their importance as platforms for publishing influential research in the VAP domain, highlighting their role in advancing understanding and treatment in this field.

Cited Author

**Figure 9:**
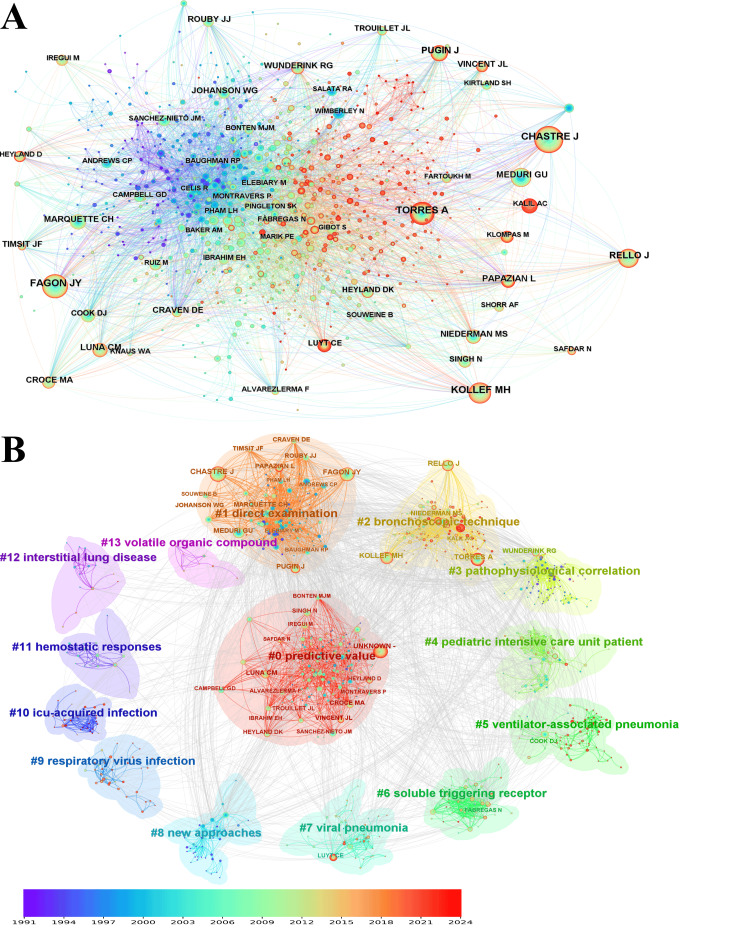
The bibliometric analysis of cited authors in the field of bronchoalveolar lavage in the diagnosis and treatment of ventilator-associated pneumonia. A, cited authors co-operation network; B, cited authors co-operation cluster. The color gradient, ranging from purple (1991) to red (2024), represents the publication year of the topics.

The findings of the bibliometric analysis of VAP research reveal significant insights into the most influential cited authors and the structure of the research network. Chastre [21] emerged as the most cited author within Cluster #1, with 453 citations. Griffin [31] is also in Cluster #1, with 393 citations. Kollef [32] in Cluster #2 received 355 citations, with Torres [25] closely behind with 344 citations, and Rello [33] rounded out the top five with 322 citations (Figure 9A). This analysis identified 15 distinct clusters, each representing a unique research focus in the field of VAP. Cluster #0, the largest, was centered on the predictive value of diagnostic tests and markers, indicating a keen interest in prognostic tools within the community. The significance of direct diagnostic approaches in VAP research is underscored by the presence of two clusters (clusters 1 and 2) dedicated to direct examination, albeit with slightly varying member counts and silhouette values. Additionally, clusters focusing on pathophysiological correlations and nuances of treating pediatric intensive care unit patients highlighted the diversity of research areas encompassing pathophysiology and pediatric care (Figure 9B).

Regarding the impact of authors, as measured by bursts of citations, Kalil [30] stood out for its significant contributions to the field. Other noteworthy contributors included Johanson [34] and Meduri [17], who were distinguished by the notable bursts of citations they garnered, reflecting the significant impact and relevance of their contributions over time (Table 5).

**Table 5:** Top 25 cited authors with the strongest citation bursts.

Cited authors	Emergence year	Strength	Begin year	End year
Johanson	1991	20.08	1991	1998
Andrews	1991	17.48	1991	2000
Salata	1991	14.26	1991	2003
Thorpe	1991	13.87	1991	2001
Kahn	1991	13.7	1991	2002
Meduri	1992	18.53	1992	2000
Wimberly	1992	17.69	1992	2003
Pham	1992	15.9	1992	2001
Decastro	1992	14.85	1992	2002
Rouby	1992	14.46	1992	1999
Guerra	1992	11.94	1992	2002
Bell	1993	14.09	1993	1999
Pingleton	1994	14.38	1994	2002
Marquette	1992	12.4	1994	2002
Niederman	1992	12.49	1995	2000
Ibrahim	2002	12.74	2002	2012
Shorr	2003	15.22	2006	2015
Gibot	2005	13.54	2006	2014
Heyland	1995	16.66	2007	2015
Safdar	2006	13.52	2007	2020
Klompas	2008	17.53	2010	2024
Luyt	2004	15.6	2011	2024
Unknown	1995	30.39	2012	2024
Melsen	2011	16.77	2014	2024
Kalil	2017	47.36	2017	2024

Centrality analysis, which assesses the influence or connectivity of authors within the citation network, identifies Wunderink [35] as the leading cited author. Centrality analyses indicated that these authors played critical roles in bridging diverse research topics within the VAP field, enhancing the development and understanding of this scientific domain.

Discussion

The application of BAL as a diagnostic and therapeutic tool in the management of VAP reveals potential areas for improvement in current clinical practice. In particular, in the selection of antibiotic treatment regimens and the evaluation of treatment outcomes, the microbiological and inflammatory marker information provided by BAL holds significant value in the formulation of customized treatment plans [4,6,7,13]. Through bibliometric analysis, we systematically reviewed the role and value of BAL in the diagnosis and treatment of VAP using a retrospective literature analysis design. By collecting and evaluating studies published over the past 20 years, this study revealed research trends, keywords, principal authors, and institutions, as well as hotspots and future directions within the field. The co-citation analysis underscores the importance of BAL technology in the diagnosis and treatment of VAP, particularly its potential to enhance diagnostic accuracy and treatment outcomes. Keyword co-occurrence and cluster analysis further identified prominent research areas such as microbiological testing, antibiotic treatment strategies, and the emerging application of biomarkers, all of which are significant for improving VAP management strategies.

Our study revealed that co-citation analysis highlighted several pioneering studies within the domain, primarily focusing on the diagnostic accuracy, treatment effectiveness, and safety assessment of BAL technology. The top three most cited articles were as follows: a study by Chastre (1995) titled “Evaluation of bronchoscopic techniques for the diagnosis of nosocomial pneumonia” published in the American Journal of Respiratory and Critical Care Medicine [19], cited 11 times; a study by Torres [12], cited 24 times, emphasizing the quantitative culture diagnosis of VAP; Meduri, GU’s work “Causes of fever and pulmonary densities in patients with clinical manifestations of ventilator-associated pneumonia” published in CHEST [36], cited 11 times. Keyword co-occurrence analysis showed that “VAP,” “bronchoalveolar lavage,” “diagnosis,” “treatment,” “microbiological testing,” and “antibiotic treatment” were the most frequently occurring keywords in the research. In recent years, “microbiological testing” and “antibiotic treatment” have become increasingly important areas of research. Author collaboration network analysis revealed several key research groups, including Chastre, Torres, and Meduri, which are the most active and productive core authors. International collaborations in this field have gradually increased, particularly among research institutions in the United States, Europe, and Asia. The bibliographic coupling network analysis indicated that the application of BAL in VAP treatment is a highly concentrated research area, with related themes including “drug sensitivity testing,” “antimicrobial drug selection,” and “efficacy evaluation.” Temporal views and cluster analyses revealed the evolution of research themes, showing shifts in research focus within the VAP domain, such as innovative applications of BAL technology, solutions to microbial resistance issues, and optimization of patient management strategies. Geographic analysis revealed the contributions of scientific institutions across various countries and regions globally to the VAP research domain, with the main research force concentrated in developed countries with advanced medical resources and research foundations. In particular, research collaborations from the United States, Germany, China, and Japan have been the most active in this field, reflecting the contributions and influence of different regions in VAP research and treatment methodology development. Moreover, the discovery of emerging technologies and methods applied in BAL offers new insights for enhancing the efficiency of VAP diagnosis and treatment.

Based on the findings of this study, future research should focus on several aspects. First, further clinical studies are necessary to verify the effectiveness and safety of bronchoalveolar lavage in the diagnosis and treatment of VAP, especially across different types of VAP patient populations. Second, exploring the combined use of bronchoalveolar lavage technology and emerging biomarker detection methods may enhance the diagnostic accuracy of VAP. Finally, with the issue of antibiotic resistance becoming more prominent, the flexible application of bronchoalveolar lavage in personalized anti-infection treatment plans offers more options for this clinical challenge.

Despite providing comprehensive insights into the role and value of bronchoalveolar lavage in the diagnosis and treatment of VAP, this study had some limitations. First, the bibliometric analysis relies on published literature, which may be subject to publication bias. Second, the interpretation of the analysis results must be integrated with actual clinical experience and expertise and should not be used in isolation to guide clinical decision-making. Finally, the scope of the study period and selection of databases may have limited the comprehensiveness of the analysis.

## Conclusions

Overall, through a bibliometric analysis, this study offers perspectives for understanding the role and value of BAL in the diagnosis and treatment of VAP. Our analysis underscores the importance of BAL in the management of VAP, particularly its potential applications in enhancing diagnostic accuracy, guiding antibiotic treatment selection, and evaluating treatment outcomes. Given the high incidence of VAP and its associated clinical challenges, strengthening research on the application of BAL technology in VAP management is crucial to improve patient prognosis and reduce healthcare costs. Combining BAL technology with advanced biomarker detection is expected to lower the risk of VAP and improve the diagnosis and treatment efficiency.
